# The Role of Histone Lysine Methylation in the Response of Mammalian Cells to Ionizing Radiation

**DOI:** 10.3389/fgene.2021.639602

**Published:** 2021-03-30

**Authors:** Elena Di Nisio, Giuseppe Lupo, Valerio Licursi, Rodolfo Negri

**Affiliations:** ^1^Department of Biology and Biotechnology Charles Darwin, Sapienza University of Rome, Rome, Italy; ^2^Institute of Molecular Biology and Pathology, National Research Counsil (IBPM-CNR), Rome, Italy

**Keywords:** DNA damage, ionizing radiation, DNA repair, HPTMs, histone methylation

## Abstract

Eukaryotic genomes are wrapped around nucleosomes and organized into different levels of chromatin structure. Chromatin organization has a crucial role in regulating all cellular processes involving DNA-protein interactions, such as DNA transcription, replication, recombination and repair. Histone post-translational modifications (HPTMs) have a prominent role in chromatin regulation, acting as a sophisticated molecular code, which is interpreted by HPTM-specific effectors. Here, we review the role of histone lysine methylation changes in regulating the response to radiation-induced genotoxic damage in mammalian cells. We also discuss the role of histone methyltransferases (HMTs) and histone demethylases (HDMs) and the effects of the modulation of their expression and/or the pharmacological inhibition of their activity on the radio-sensitivity of different cell lines. Finally, we provide a bioinformatic analysis of published datasets showing how the mRNA levels of known HMTs and HDMs are modulated in different cell lines by exposure to different irradiation conditions.

## Introduction

The first and basic level of chromatin organization consists in the wrapping of genomic DNA around histone octamers forming the nucleosomes (Luger et al., 2012). Nucleosomes mediate the interactions of the genomic DNA with all the effectors involved in fundamental biological processes such as transcription, replication, recombination, damage response and repair (Groth et al., 2007; McGinty and Tan, 2015). Histone tails, which protrude from the nucleosome core, are the target of a plethora of post-translational chemical modifications, such as: acetylation, methylation, phosphorylation, ubiquitylation, sumoylation and ADP-ribosylation (Campos and Reinberg, 2009; Bannister and Kouzarides, 2011). These Histone Post-Translational Modifications (HPTMs) form a sophisticated code of signals, known as histone code, which regulates the interactions of the genome with very important cellular effectors involved in several biological processes. The HPTMs are fixed on the histone tails by **writers** and can be reversed by **erasers.** The histone code is then interpreted by **readers,** proteins containing a domain that binds to the modified histone residue with high specificity (Yun et al., 2011). Once locally recruited, the readers can promote further histone modifications, structural changes in naked DNA or nucleosomal structure, or the recruitment of another protein function. Histone methylation, in particular, plays a key role in transcriptional regulation (Venkatesh and Workman, 2015) and DNA damage repair (Lukas et al., 2011; Gong and Miller, 2019). Mono-, di-, and tri-methylation can be catalyzed by specialized histone methyltransferases (HMTs) at specific lysine (K) and arginine (R) residues on histone tails and erased by specific histone demethylases (HDMs). In mammalians, the canonical lysine methylation sites are found on histone H3 tail at lysine 4 (H3K4), lysine 9 (H3K9), lysine 27 (H3K27), lysine 36 (H3K36) and lysine 79 (H3K79), and on histone H4 tail at lysine 20 (H4K20) (Husmann and Gozani, 2019). These modifications regulate an array of chromatin functions with different outcomes, depending from the specific modified residue. There are additional, though less characterized, sites of lysine methylation, such as H3K23, H3K63, H4K5, and H4K12. Moreover, different histone variants can undergo methylation of specific lysine residues, such as H2AXK134, with an important impact on damage response regulation (Sone et al., 2014). In humans, two annotated protein domains carry out lysine methylation: the SET domain (named after three *Drosophila melanogaster* proteins originally recognized as containing this domain, namely Su(var)3–9, Enhancer of Zeste and Trithorax), and the seven-beta-strand (7βS) domain (which is characteristic of the non-SET-domain enzymes, including the histone KMT hDOT1L and DNA methyltransferases) (Husmann and Gozani, 2019). The two families together account for more than 200 enzymes with different amino-acidic residue specificity and expression levels in different cell types (Husmann and Gozani, 2019). In the case of HDMs, two large groups have been identified in eukaryotes: the LSD1 family and the Jumonji C-domain-containing family. LSD1, belonging to the flavin adenine dinucleotide-dependent amine oxidase superfamily, is the first identified histone demethylase (Shi et al., 2004). Jumonji C-domain containing HDMs (JHDMs) are Fe2+ and α-ketoglutarate-dependent hydroxylases. They are divided into 7 phylogenetically distinct subfamilies (JHDM1-3, JARID, PHF2/PHF8, UTX/UTY, JmjC domain only), each sharing a common set of substrates (Tsukada et al., 2006; D’Oto et al., 2016). An alternative classification of HDMs in 7 groups (KDM1-6 clusters and PHF cluster, also known as KDM7 group), based on sequence homology, on architecture of associated motifs and on histone modification substrates, is also commonly used (Pedersen and Helin, 2010) and will be adopted in this review. Some excellent reviews on the role of histone and non-histone methylation in genotoxic damage sensing and repair were previously published (Chen and Zhu, 2016; Wei et al., 2018; Gong and Miller, 2019). In the next paragraphs, we will review recent progress in this field, focusing on histone lysine methylation changes associated with the genotoxic damage induced by ionizing radiation (IR) and on their role in triggering damage checkpoint and DNA repair specifically in mammalian cells. We will also discuss the role of specific HMTs and HDMs in these processes and the effects caused by the modulation of their expression or by the pharmacological inhibition of their activity. Finally, we present a bioinformatic analysis of published datasets showing that IR modulates HMTs and HDMs expression in different mammalian cell types and tissues.

## The Cell Response to IR-Dependent Damage

IR causes cell damage by either a direct or an indirect action on biological molecules, such as nucleic acids, proteins and lipidic membrane components. The former action involves the radiation directly absorbed by the biological molecules, whereas the indirect action is mediated by free radicals, such as hydroxy, alkoxy and peroxy radicals, generated by the interaction of the radiation with water molecules (Desouky et al., 2015). Additional indirect damage may involve reactive nitrogen species (RNS) or ionized atoms in targeted biomolecules (Wardman, 2009). The balance between direct and indirect damage may consistently change depending on the radiation quality. X-ray and γ-ray photons deposit energy in a highly dispersed manner, defined as low Linear Energy Transfer (LET), thus causing mainly indirect damage. In contrast, heavily charged particles are characterized by high LET, are densely ionizing and penetrate cells and tissues in straight trajectories (Durante, 2004). Mammalian cells respond to IR by a complex strategy, which aims to activate the following three main countermeasures to the cellular damage:

(a) Slowing down or even blocking the cell cycle to give the cellular apparatus the time to repair the damage before proceeding with replication of the genetic material and/or cell division;
(b) Activating mechanisms of protection from damage (i.e., hydroxyl radical scavengers) and genome repair systems;
(c) Activating apoptotic programs in case of irreparable damage;

This complex response is based on a series of damage sensors activating a sophisticated system of signal transduction, which in turn triggers downstream effectors (Bartek and Lukas, 2007). Ultimately, the response requires a reprogramming of the cell transcriptome, in order to modulate the expression of a plethora of genes involved in response-dependent pathways. The most important component of the system is the DNA damage response (DDR), triggered by sensors of genomic damage, whereas little is known about pathways sensing the damage to other macromolecules. Exposure of cells to IR produces genotoxic damage consisting of single-strand breaks (SSBs), base lesions (i.e., purine oxidation) and double-strand breaks (DSBs). These lesions can occur at genomic sites individually or in combination (clustered damage), depending on radiation features (dose, dose rate, quality of radiation, LET) (Mavragani et al., 2019). Genome integrity is continuously checked by the DDR, a series of complex interacting cellular pathways triggered by the sensor protein complexes located at the genomic lesions (Polo and Jackson, 2011).

### The Histone Methylation Landscape at the Damaged Genomic Sites

**FIGURE 1:**
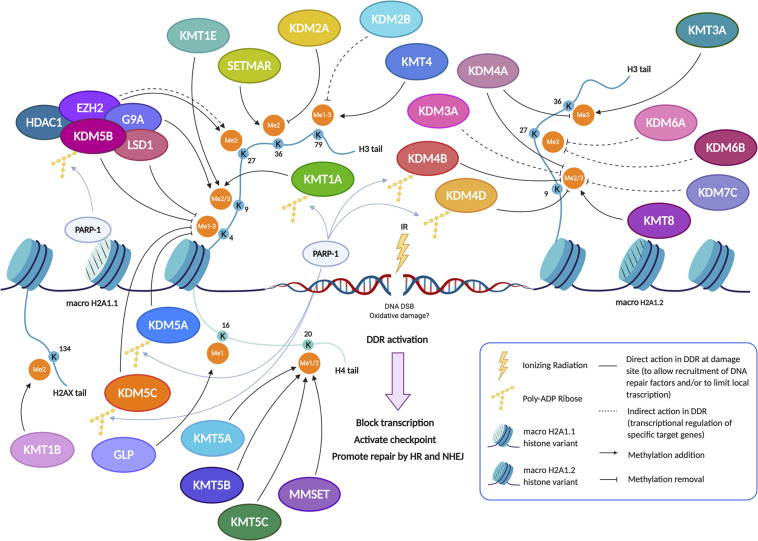
Pre-existing chromatin features and damage-induced histone modifications regulate the DNA repair pathways. The set of changes of HPTMs coordinately orchestrates the DDR around the damaged site, reorganizing chromatin structure in order to limit local transcription and to promote the DNA repair through the recruitment, the action and the retention of sensors, transducers and effectors of damage signaling cascade. In particular, methylation dynamics at different lysine residues, preferentially on H3 and H4 histone tails, regulate the response to DNA damage both temporally and spatially. Indeed, several histone methylation changes occur on H3K4, H3K9, H3K27, H3K36, H3K79, H4K16, H4K20, and H2AXK134 to aid in DNA damage repair. Lysine methylation is a highly dynamic histone modification owing to the interplay between KMTs (Lysine methyltransferases) and KDMs (Lysine demethylases). After the induction of genotoxic damage by ionizing radiation (IR), several KMTs and KDMs promote the formation of a chromatin landscape prone to DNA repair, acting in synergy or antagonistically to favor the HR and/or NHEJ pathway. Indeed, some demethylases and methyltransferases act at DSB sites, thus directly regulating the recruitment of DDR factors; the demethylase/methyltransferase activity of these epigenetic modifiers modulates the DDR not only through a direct role (non-dashed lines), but also indirectly (dashed lines) by regulating the expression of such DSB repair factors, acting at their promoter regions. The recruitment of some of these enzymes, including KDM5B, is specific and requires the presence of histone variant macroH2A1.1 and PARylation by PARP1. In other cases, such as KMT8, the recruitment arises thanks to the interaction with the histone variant macroH2A1.2 without PARylation; alternatively, the recruitment can take place thanks to reader domains which recognize specific histone marks (see also Figure 3). Created using BioRender.

Damage signaling and repair are operated in the context of chromatin structure with strict requirements for full efficiency (Polo and Jackson, 2011). A number of histone modifications have been associated to specific lesions and their functional role in damage signaling and repair has been postulated (Vidanes et al., 2005; Misteli and Soutoglou, 2009; Wei et al., 2018; Husmann and Gozani, 2019). HMTs and KDMs can directly modify the chromatin at DNA damaged sites to promote damage sensing, to favor the recruitment of DNA repair proteins, to silence transcription during the repair process, to regulate the expression of DDR factors and to change and restore the chromatin state before and after repair. Indeed, lysine methylation is one of the prominent histone modifications at damaged sites. It mainly occurs at lysines on H3 tails, but methylation at H4K20 and K16 and at H2AXK134 are also relevant (Figure 1). In the next sections, we will summarize what it is currently known on the role of the methylation state of specific histone lysines in signaling IR-induced damage and promoting DNA repair, and on the modification machinery involved in this methylation.

## Lysine Methylation in Histone H3 Tail

### H3K4

**FIGURE 2:**
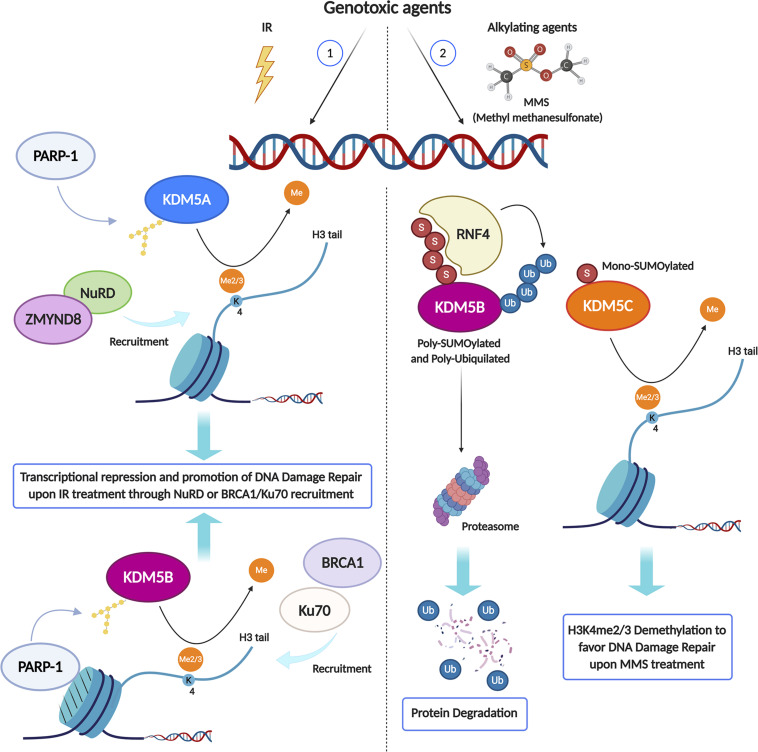
KDM enzymes may play different roles in the DNA Damage Response (DDR) depending on the genotoxic agent type. Upon IR treatment (1), PARP1 activity is required to recruit both KDM5A and KDM5B at DSB sites, where they catalyze the local demethylation of H3K4me2/3, crucial for the recruitment of transcriptional repressive complex like ZMYND8/NuRD or DDR key proteins, including BRCA1 and Ku70. Conversely, in response to alkylation damage by MMS (2), Poly-SUMOylated KDM5B is Poly-ubiquitylated by the SUMO-targeted ubiquitin ligase RNF4 and degraded by the proteasome, while JARID1C is Mono-SUMOylated and recruited to the chromatin to demethylate histone H3K4me2/3. Created using BioRender.

**FIGURE 3:**
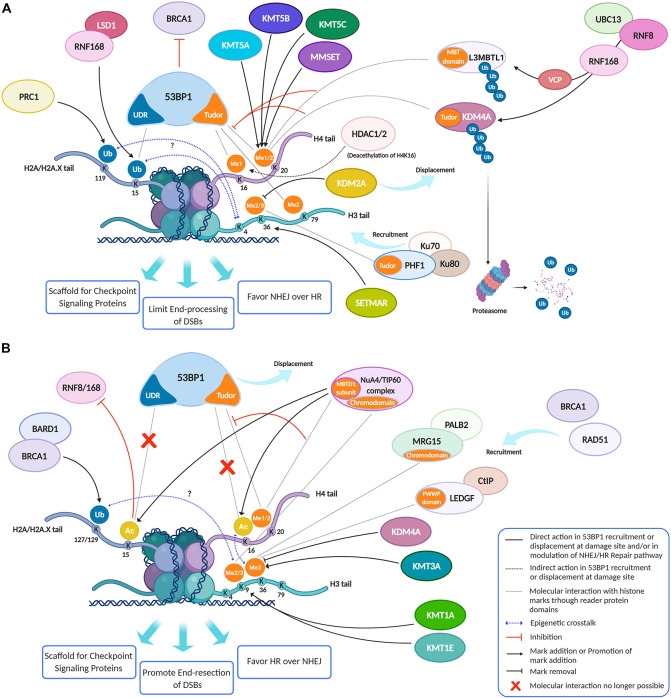
Histone modifications, dynamically added or removed around the DNA damage site, constitute an integrated signal for the DSBs repair pathway choice. **(A)** 53BP1 is a master regulator of DNA double-strand break repair pathway choice toward NHEJ and its binding to chromatin is mainly regulated by specific histone modifications, comprising H2A ubiquitylation and H3 and H4 methylation. Indeed, specific HPTMs can generate a docking site for 53BP1, which binds the ubiquitinated H2A/H2A.X K13/15/119 through its UDR motif and H4K20me2, H4K16me1, and H3K79me2 through its tandem Tudor domain. Therefore, despite H4K20me2 being the main histone mark involved in 53BP1 recruitment, other marks may contribute to it. For example, the deacetylation of H4K16 by HDAC1/2 indirectly promotes the 53BP1 recruitment and the NHEJ over HR, because it makes possible the methylation of this lysine residue. Under normal conditions, the H4K20me2 mark is masked by various interacting proteins, including L3MBTL1 and KDM4A/JMJD2A. Upon DNA DSBs induction, the ATM phosphorylation cascade promotes the recruitment of the E3 ubiquitin ligase RNF8 to the DNA damage site, which, together with the E2 ubiquitin ligase UBC13, promotes the relocation of the E3 ubiquitin ligase RNF168 to the DNA break sites. LSD1 may favor the 53BP1 recruitment interacting with RNF168 and/or thanks to a possible epigenetic cross-talk between H3K4 demethylation and H2A ubiquitylation. RNF168 catalyzes in turn the H2A K13/15 mono-ubiquitination and the poly-ubiquitination of L3MBTL1 and KDM4A, leading to RNF8-dependent degradation of KDM4A and to VCP-mediated and ubiquitin-dependent removal of L3MBTL1 from chromatin, favoring the unmasking of H4K20me2 and the loading of 53BP1 around the damaged site. LSD1 can also disadvantage 53BP1 recruitment, negatively regulating the interaction between p53 and 53BP1 (not shown). After DSB induction by IR, the H3K36me2 markedly increases, improving the association of early NHEJ factors such as Ku70/Ku80 and NBS1 at the damage site. Once activated, ATM (not shown) may negatively regulate KDM2A chromatin-binding capacity, favoring its displacement, which favors an increase of H3K36 di-methylation mark near the DNA damage sites by SETMAR. H3K36me3 can favor the retention of the NHEJ-associated factor PHF1, which is recruited in a Ku70/Ku80 dependent way and which supports an open chromatin for the efficient DNA repair. In contrast to H3K36me2, H3K36me3 regulates both NHEJ and HR, probably depending on the relative abundance of its reader factors and/or by the chromatin context. In this regard, pre-existing transcription-associated and cell cycle-regulated histone modifications together with those damage-induced regulate the DNA repair pathways. **(B)** The pre-existing H3K36me3 mark at actively transcribed gene bodies favors DNA repair by HR pathway choice: in human cells, both LEDGF (Lens epithelium-derived growth factor), via its PWWP reader domain, and MRG15, via its chromodomain, can bind H3K36me3, promoting the recruitment of CtIP and PALB2, two crucial factors for the DNA end-resection and strand invasion, respectively. Moreover, mutually exclusive histone modifications can contribute to tip the balance between HR and NHEJ in DSB repair pathway choice: a switch between ubiquitylation and acetylation on H2AK15 and H4K16 can favor HR over NHEJ. The human NuA4/TIP60 acetyltransferase complex is able to inhibit the 53BP1 recruitment both through this mechanism and by targeting the same H4K20me2 mark, by binding H4K20me with the MBT (malignant brain tumor) domain of MBTD1, its stable subunit. Moreover, the human NuA4/TIP60 acetyltransferase complex promotes the repositioning of 53BP1 through other mechanisms, such as the acetylation of H2A/H2AX K13/15 that blocks the recruitment of RNF8/168 and through the acetylation of H4K16 that impedes the methylation of this lysin residue, limiting the recruitment of 53BP1. KDM4A, besides masking H3K20me2 and limiting the 53BP1 recruitment thus inhibiting the NHEJ pathway, negatively regulates the DDR also removing H3K36me3 mark thus inhibiting the HR pathway. Conversely, KMT3A favors the HR through the methylation of H3K36. KMT1E favors the HR though the methylation of H3K9. Indeed, the H3K9me2 mark is involved in the recruitment of NuA4/TIP60 acetyltransferase complex, and H3K9me3 is involved in the recruitment of BARD1/BRCA1 Ub ligase activity on H2AK127 favoring the DNA end-resection, the repositioning of 53BP1 and thus the HR. Created using BioRender.

Strong evidence in the literature shows that lysine methylation in histone H3 tail is implicated in damage signaling. Chromatin changes are required in order to silence transcription *in cis* to DNA double-strand breaks (Shanbhag et al., 2010). Since H3K4 tri-methylation is associated with active transcription genome-wide (Vakoc et al., 2006), active demethylation of this residue around damaged sites is expected. Indeed, Li and coworkers (Li et al., 2014) found that KDM5B, a HDM specifically involved in H3K4me3 demethylation, is redistributed in the nucleus after exposure to IR and becomes highly enriched at the damaged sites. The mechanism of KDM5B recruitment is quite specific and requires the presence of nucleosomes containing histone variant MacroH2A1.1 and PARylation by PARP1 (Figure 1). KDM5B is required for efficient homologous recombination (HR) and non-homologous end joining (NHEJ) and, specifically, for the recruitment of Ku70 and BRCA1 to IR-induced breaks (Li et al., 2014). KDM5B is upregulated in several tumors and cancer-derived cell lines, which seems to be associated with resistance to genotoxic stress (Xu et al., 2018). Moreover, microRNAs that specifically target KDM5 mRNA are downregulated in tumors and, when overexpressed in cancer cells, increase their radio-sensitivity (Mocavini et al., 2019). KDM5B associates with several other proteins with transcription repressing activities, such as the HDM LSD1, the histone deacetylase HDAC1 and the histone methylase EZH2 (Figure 1). This raises the question of the respective functional roles of the H3K4me3 demethylating activity and of the corepressor recruiting activity of KDM5B. In this respect, it was recently shown that inhibition of KDM5B demethylase activity increases the radio-sensitivity of cancer cell lines upregulating KDM5B, suggesting that the catalytic activity of KDM5 is important in this context (Bayo et al., 2018; Pippa et al., 2019). Two other class 5 HDMs, KDM5A and KDM5C, both specific for H3K4me3, have also been involved in radio-resistance (Hendriks et al., 2015; Gong et al., 2017, Figures 1, 2). Gong and coworkers showed that KDM5A performs H3K4me3 demethylation within chromatin near DSBs and has a crucial role in regulating the ZMYND8-NuRD chromatin remodeler (Gong et al., 2017), which represses transcription and promotes DNA repair (Gong et al., 2015). H3K4me3 must be demethylated to allow repair to proceed, probably because this process is essential for limiting local transcription. The removal of methylation marks is therefore just as important as the addition of new histone modifications (Price, 2017). While KDM5A seems to act in parallel to KDM5B, although with different mechanisms, KDM5C could be recruited in alternative circumstances. Hendriks and coworkers showed that, in response to alkylation damage by methyl methanesulfonate (MMS), SUMOylated JARID1B (KDM5B) is ubiquitylated by the SUMO-targeted ubiquitin ligase RNF4 and degraded by the proteasome, whereas JARID1C (KDM5C) is SUMOylated and recruited to the chromatin to demethylate histone H3K4 (Hendriks et al., 2015). This observation suggests that the redundancy among HDMs could be exploited to differentiate the response pathways triggered by different genotoxic agents (Figure 2). KDM5B is also a physical component of the LSD1/NuRD complex, which functions in transcriptional repression. LSD1 (KDM1A) specifically demethylates H3K4 me1/2 (Shi et al., 2004). Although KDM5B and LSD1 in the complex can act in a sequential and coordinated manner to demethylate H3K4 (Li et al., 2011), the role of LSD1 in DDR may be partially distinct from that of class 5 HDMs. LSD1 can be recruited directly to sites of DNA damage, possibly through the interaction with the E3 ubiquitin ligase RNF168, thus reducing H3K4me2 levels (Mosammaparast et al., 2013). However, since CK2-mediated phosphorylation of LSD1 promotes the molecular interaction between RNF168 and 53BP1, some of LSD1 roles in DDR may be independent of its catalytic activity (Peng et al., 2015). Although the loss of LSD1 does not affect the initial formation of γ-H2AX foci, 53BP1 and BRCA1 complex recruitment appears to be reduced upon LSD1 knockdown (Mosammaparast et al., 2013). This work suggests that LSD1 affects 53BP1 recruitment by promoting H2A ubiquitylation at damaged sites during late S/G2. This could be mediated by the interaction of LSD1 with RNF168 and/or by a possible crosstalk between H3K4 demethylation and H2A ubiquitylation (Figure 3A). Once again, it is likely that one function of LSD1-mediated demethylation in this pathway is to facilitate a repressive chromatin environment near DSBs, which may silence transcription near these sites. Consistent with a role in DDR, knockdown of LSD1 results in moderate hypersensitivity to γ-irradiation, although, paradoxically, the efficiency of HR appears increased (Mosammaparast et al., 2013; Wojtala et al., 2019). To explain this ambivalent effect, it should be considered that LSD1 can also promote p53 demethylation, thereby inhibiting p53 function in DDR activation (Huang et al., 2007).

### H3K9

Coherently with the requirement of a repressive chromatin environment, DNA damage promotes methylation of histone H3 on lysine 9 (Sun et al., 2009; Ayrapetov et al., 2014; Khurana et al., 2014). Tri-methylated H3K9 is strongly associated with transcriptional repression (Campos and Reinberg, 2009; Bannister and Kouzarides, 2011). X-ray-induced repositioning of γ-H2AX overlaps with histone H3K9me3 heterochromatin marks in the nuclei of irradiated cells (Hausmann et al., 2018). A complex containing the H3K9 methyltransferase KMT1A (SUV39H1) is rapidly loaded onto the chromatin at DSBs induced by laser micro-irradiation, spreading tens of kilobases away from DSBs (Hausmann et al., 2018). The role of H3K9 methylation at the site of damage goes beyond transcriptional repression, since it is required to activate the Tip60 acetyltransferase, allowing Tip60 to acetylate both ataxia- telangiectasia mutated (ATM) kinase and histones H4 and H2A (Sun et al., 2009; Ayrapetov et al., 2014). Histone acetylation creates local regions of open and relaxed chromatin, which facilitate DSB repair and compete with the binding of 53BP1 to damaged chromatin. Since 53BP1 inhibits DNA ends resection and promotes NHEJ-mediated repair, H3K9 methylation can favor HR over NHEJ (Tang et al., 2013; Jacquet et al., 2016; Gursoy-Yuzugullu et al., 2017; Figures 3A,B). Cells lacking KMT1A, which was previously implicated in genome stability (Peters et al., 2001; Peng and Karpen, 2009), display defective activation of Tip60 and ATM, decreased DSB repair, and increased radio-sensitivity (Sun et al., 2009; Ayrapetov et al., 2014). Other H3K9 HMTs have been implicated in DDR (Figure 1). KMT8 (PRDM2) is recruited to damage chromatin sites through histone MacroH2A1.2 and contributes to H3K9 methylation (Khurana et al., 2014). KMT1E (SETDB1) is also enriched at damaged sites, where it promotes 53BP1 repositioning and HR (Alagoz et al., 2015). In contrast, KMT1C (G9A), which can interact with KDM5B-LSD1, may have an inhibitory role on DDR. This protein is specifically degraded by the proteasome in response to DSBs (Takahashi et al., 2012) and its downregulation was recently shown to trigger DDR in colorectal cancer cells (Zhang et al., 2015). KMT1C is not entirely specific for H3K9, since it can methylate H3K27 and has some non-histone substrates related to DDR, such as p53 (Huang et al., 2010). In agreement with a key role of H3K9 methylation in DDR, the G9A inhibitors UNC0638 and A-366 hypersintetized tumor cells to low doses of DSB-inducing agents (Agarwal and Jackson, 2016); moreover, Price and coworkers showed that the G9A specific inhibitor BIX-01294 can significantly increase tumor cell sensitivity to ionizing radiation (Gursoy-Yuzugullu et al., 2017). H3K9-specific HDMs were also associated with DDR, suggesting that H3K9 methylation could play a dynamic role in DDR (Young et al., 2013; Khoury-Haddad et al., 2014; Figure 1). In particular, KDM4D is rapidly recruited to laser micro-irradiated genomic regions through a PARP-mediated process and is required for efficient formation of IR-induced foci of Rad51 and 53BP1 and for ATM recruitment. A catalytic deficient KDM4D cannot rescue the phenotypes caused by KDM4D knockdown (Khoury-Haddad et al., 2014), showing that the demethylase activity of KDM4D is required for DDR regulation. This was confirmed by using 8-hydroxyquinoline (8-HQ), a catalytic inhibitor of KDM4D (Khoury-Haddad et al., 2014). KDM4B is also selectively recruited via PARP1 to sites of IR-dependent damage, and its overexpression increases the kinetics of γ-H2AX foci resolution and confers a survival advantage following γ-irradiation (Young et al., 2013). Conversely, Fan et al. (2020) found that the histone demethylase KDM3A (JMJD1A) positively regulates the expression of DDR genes by promoting both the expression of c-Myc and its chromatin recruitment through H3K9 demethylation, suggesting a new possible target to sensitize prostate cancer to radiotherapy (Fan et al., 2020). KDM7C (PHF2) is a new H3K9 demethylase, which positively regulates HR-mediated repair through the transcriptional control of CtIP and BRCA1 by direct demethylation of H3K9me2 at their promoters (Alonso-de Vega et al., 2020).

### H3K27

Methylation of H3K27 has been observed at DSB sites (O’Hagan et al., 2008; Chou et al., 2010) or following oxidative damage (Campbell et al., 2013). H3K27 methylation may contribute to PARP-dependent transcriptional repression by removing elongating RNA polymerase II from sites adjacent to DNA lesions and promoting recruitment of subunits of the Polycomb repressive complexes PRC1 (Chou et al., 2010) and PRC2 (O’Hagan et al., 2008, 2011; Chou et al., 2010; Campbell et al., 2013) to damaged sites. PRC2 produces H3K27me2 and H3K27me3 via EZH1 and EZH2 (Simon and Kingston, 2009). Enrichment of EZH2 (KMT6) has been observed near DSBs induced by I-SceI endonuclease in epithelial cells (O’Hagan et al., 2008) or DNA lesions caused by UV laser micro-irradiation (Chou et al., 2010; Campbell et al., 2013) and H_2_O_2_ treatment, but not at IR-induced foci (O’Hagan et al., 2011). EZH2 could also be indirectly involved in DDR by transcriptional regulation of specific genes after DNA damage (Wu et al., 2011). Moreover, EZH2 localizes at stalled replication forks, where it increases H3K27me3 levels, mediating recruitment of the MUS81 nuclease to promote replication fork restart (Rondinelli et al., 2017). Indeed, low EZH2 levels reduce H3K27 methylation and prevent MUS81 recruitment at stalled forks, causing fork stabilization (Rondinelli et al., 2017). Although shRNAs targeting EZH2 or its chemical inhibitor GSK126 significantly promoted genotoxicity and increased cell chemosensitivity (Gao et al., 2017), GSK126 treatments did not enhance the sensitivity of glioma cells to γ-irradiation (Gursoy-Yuzugullu et al., 2017), suggesting that the role of EZH2 could be dependent on DNA damage quality. As in the case of H3K9, H3K27-specific HDMs have also a role in DDR, suggesting that a dynamic equilibrium between methylation and demethylation is required. Irradiation of tumor cells was shown to result in a rapid loss of H3K27me3, which was prevented by the siRNA-mediated knockdown of the H3K27 demethylase KDM6A (UTX) (Rath et al., 2018). Knockdown of UTX enhanced the radio-sensitivity of tumor cell lines. Treatment of tumor cells with the UTX demethylase inhibitor GSKJ4 shortly before irradiation prevented the IR-induced reduction in H3K27me3, enhancing radio-sensitivity (Rath et al., 2018; Katagi et al., 2019). As determined by comet assay and γH2AX expression levels, this GSKJ4 treatment protocol inhibited the repair of IR-induced DSBs. Consistently with *in vitro* results, treatments of mice bearing leg tumor xenografts with GSKJ4 enhanced IR-induced tumor growth delay (Rath et al., 2018). In contrast with results obtained in tumor cell lines, IR had no effects on H3K27me3 levels in normal fibroblast cell lines and GSKJ4 treatments did not increase their radio-sensitivity. Overall, these data suggest that H3K27me3 demethylation plays a role in DSB repair in tumor cells and that the UTX demethylase may be a target for the selective radio-sensitization of tumor cells (Rath et al., 2018). As in the case of EZH2, the effects of H3K27-HDMs on radio-sensitivity could be indirect and mediated by the transcriptional regulation of target genes. Indeed, upon exposure to IR, KDM6A (UTX) in *Drosophila* and KDM6B (JMJD3) in mammalian cells are recruited to promote the expression of specific genes involved in DNA repair, including Ku80, in a p53-dependent manner (Zhang et al., 2013; Williams et al., 2014; Katagi et al., 2019).

### H3K36

Histone H3K36 methylation is well-known for its role in transcriptional regulation (Chen et al., 2020). H3K36me2 and me3 are hallmarks of active transcription preferentially located in the 5′ and 3′ portion of gene bodies, respectively. H3K36 methylation has also been linked to multiple biological processes, including transcriptional silencing, alternative splicing, DNA replication, as well as DDR and repair (Chen et al., 2020). H3K36me2 deposition is rapidly induced globally and locally after IR and DSB induction (Fnu et al., 2011; Aymard et al., 2014). Di-methylation of H3K36 improves the association of early DNA repair components, including Ku70/Ku80 heterodimers, with the IR-induced DSBs, and enhances DSB repair. The DNA repair protein METNASE (SETMAR), which has a SET histone methylase domain, was thought to be required for NHEJ, by localizing at DSBs and directly mediating the formation of H3K36me2 near them (Fnu et al., 2011; Figures 1, 3A). A recent study, however, suggests that the SET domain in full length SETMAR protein has lost its function in NHEJ DNA repair, possibly because its activity may be inhibited by the dimerization of the protein mediated by the MAR domain (Tellier and Chalmers, 2019).

Several studies revealed the function of H3K36me3 in recruiting RAD51 in active transcription-associated HR (Daugaard et al., 2012; Pfister et al., 2014), although H3K36me3-enriched heterochromatin is also mainly repaired by HR (Beucher et al., 2009). Interestingly, no increase of H3K36me3 levels is detected at IR-induced DSBs, indicating that pre-existing H3K36me3 is involved in RAD51 recruitment (Aymard et al., 2014; Pfister et al., 2014). In the absence of DNA damage factors, Lens epithelium-derived growth factor (LEDGF) masks H3K36me3 marks (Daugaard et al., 2012). Following IR, LEDGF promotes the recruitment of CtIP at DSBs to drive the end processing for RAD51 loading, which is necessary for HR repair (Daugaard et al., 2012; Figure 3B). HR appears as the preferred pathway for repairing DSBs located in transcriptionally active genes and H3K36me3-enriched loci, whereas inactive genes and H3K36me3-depleted regions mostly undergo NHEJ-mediated repair (Aymard et al., 2014). Coherently, depletion of SETD2 (KMT3A), the main H3K36me3-specific HMT, severely impedes HR at DSBs (Aymard et al., 2014). Furthermore, SETD2 depletion in HT1080 fibrosarcoma cells caused similar effects to RAD51 depletion, impairing DNA repair at I-SceI-induced DSBs and increasing the frequency of deletions arising from microhomology-mediated end-joining (Pfister et al., 2014). SETD2 is also required for activation of the p53-mediated checkpoint (Carvalho et al., 2014). Several studies have described the functions of H3K36-specific KDMs, such as KDM2A and KDM4A, in regulating DDR and repair (Figure 3). In particular, an ambivalent function of KDM2A in DNA damage repair was described. On the one hand, KDM2A reduces the accumulation of H3K36me2, which promotes MRE11 complex recruitment at the DSBs (Fnu et al., 2011); indeed, ATM-mediated KDM2A phosphorylation, in response to DSBs, abrogates its chromatin-binding capacity, promoting H3K36me2 accumulation near DNA damage sites and NHEJ (Figure 3A; Cao et al., 2016). Coherently, KDM2A overexpression decreases the association of early NHEJ repair components with DSBs and their repair (Fnu et al., 2011). On the other hand, KDM2A is recruited to DSBs through its demethylase activity and its zinc finger domain and stimulates 53BP1 ubiquitylation and recruitment at damaged sites (Bueno et al., 2018); indeed, KDM2A depletion or disruption of its zinc finger domain causes micronuclei’s accumulation following IR treatment. Moreover, irradiated KDM2A-deficient cells show premature exit from the G2/M checkpoint (Bueno et al., 2018). Unlike KDM2A, KDM4A negatively regulates both NHEJ and HR, through two different mechanisms. By acting as a reader of H4K20me2 (Huang et al., 2006), an important docking site for 53BP1, KDM4A regulates DNA repair by restraining 53BP1 recruitment at damaged sites (Mallette et al., 2012; Figure 3A). By acting as an eraser of H3K36me3, KDM4A impairs a proper HR (Pfister et al., 2014; Figure 3B). RNF8-dependent degradation of KDM4A reduces its binding to H4K20me2, allowing the exposure of this mark and facilitating the recruitment of 53BP1 to DSBs (Huang et al., 2006; Mallette et al., 2012). 53BP1 is a key protein in DDR (Panier and Boulton, 2014). It acts as a molecular scaffold which recruits additional DSB responsive proteins to damaged chromatin, promoting checkpoint signaling and the context-dependent selection of HR or NHEJ repair pathways (Panier and Boulton, 2014). 53BP1 and BRCA1 opposing activities influence the choice between these pathways for DBS repair (Densham et al., 2016; Figure 3).

### H3K79

H3K79 methylation has been involved in the regulation of telomeric silencing, cellular development, cell-cycle checkpoint, DNA repair, and transcriptional regulation (Farooq et al., 2016). A unique evolutionarily conserved enzyme, KMT4 (DOT1, DOT1L), is responsible for all forms of H3K79 methylation (mono-, di- and tri-methylation) in eukaryotes. H3K79 methylation is accurately regulated by a crosstalk with other histone modifications, namely H3K4 methylation and H2B ubiquitylation (Farooq et al., 2016). Although it has been suggested that KDM2B is a histone H3K79 demethylase (Kang et al., 2018), the turnover rate of this modification on a global scale is slow and likely to be dependent on the cell cycle. Indeed, H3K79 demethylation by KDM2B contributes to timely cell cycle progression by facilitating correct DNA replication through the regulation of PCNA dissociation from chromatin (Kang et al., 2020). Moreover, the early recruitment of KDM2B into the ternary complex FBXL10/RNF2/RNF68 promotes DDR (Rona et al., 2018), but the detailed mechanism of this effect remains to be investigated, also considering that there are different protein isoforms of KDM2B and of its paralog KDM2A, which could be related to different biological functions (Vacík et al., 2018). So far, the role of H3K79 methylation in DDR has been mainly related to its ability to target 53BP1 to DSBs (Huyen et al., 2004; Figure 3A). *In vitro*, 53BP1 tandem Tudor domain specifically binds to methylated H3K79 and is required for 53BP1 recruitment to DSBs, which is inhibited by the suppression of KMT4 function. Since the methylation levels of H3K79 remained unchanged after the induction of DNA damage, 53BP1 may detect DSBs indirectly through changes in higher order chromatin structure, which expose 53BP1 binding sites (Huyen et al., 2004). Docked 53BP1 at damage sites may, in turn, recruit additional proteins to activate the checkpoint response. Studies in yeast suggest that the interaction between methylated H3K79 and Rad9 (the human ortholog of 53BP1) may inhibit single-strand DNA production, representing a functional and physical barrier to end processing both at DSBs sites and uncapped telomeres (Lazzaro et al., 2008). H3K79me2 is also involved in Rad51 foci formation. Indeed, Bre1 deficiency impairs HR-mediated repair and cell cycle checkpoint response to IR-dependent damage by specifically reducing H3K79 dimethylation in mammalian cells, possibly as a consequence of the downregulation of HR-related genes (Chernikova et al., 2010). In colorectal cancer cells, KMT4 depletion or its catalytic inhibition modulate chemotherapy responsiveness, due to its role in DDR and HR pathways (Kari et al., 2019). KMT4-mediated H3K79 methylation also plays a critical role in repairing ultraviolet (UV)-induced DNA damage in yeast (Bostelman et al., 2007; Boudoures et al., 2017) and mammalian cells (Zhu et al., 2018).

## Lysine Methylation in Histone H4 Tail

The involvement of histone H4 tail methylation in DSB repair is mainly linked to 53BP1recruitment, which, in turn, is emerging as a key step in DDR pathway selection and activation (Figure 3; Mirza-Aghazadeh-Attari et al., 2019). In the absence of DNA damage, H4K20me2 is bound by the Polycomb component L3MBTL1 or by KDM4A, each of them competing with 53BP1 for H4K20me2 binding (Lee et al., 2008; Acs et al., 2011; Mallette et al., 2012). Following DSB formation, RNF8 and RNF168 promote the ubiquitin-dependent removal and degradation of L3MBTL1 and KDM4A from chromatin, and SETD8 and MMSET (KMT3G) HMTs increase the level of H4K20 mono- and di-methylation, respectively (Acs et al., 2011; Pei et al., 2011; Gursoy-Yuzugullu et al., 2017; Figure 3A). Through its Tudor domain, 53BP1 can then bind as a dimer to nucleosomes that are both ubiquitylated at H2AK15 (Fradet-Turcotte et al., 2013) and methylated at H4K20. 53BP1 recruits its downstream effectors RIF1 and MAD2L2 (REV7) to counteract BRCA1-mediated DSB relocation and promotes NHEJ in G1-phase cells (Panier and Boulton, 2014; Gupta et al., 2018). 53BP1 affinity for H4K20me2 is increased by concurrent deacetylation of H4K16 by HDAC1 and HDAC2 (Hsiao and Mizzen, 2013; Figure 3A). In *Drosophila*, a catalytic inhibitor of SUV4-20 enzymes inhibited 53BP1 association at IR-induced DSBs and specifically impaired NHEJ-mediated repair (Li et al., 2016). A-196, a potent and selective inhibitor of SUV420H1 and SUV420H2 has highlighted the importance of H4K20 methylation for genomic integrity in human cells (Bromberg et al., 2017). Consequently, depletion of PR-Set7, an HMT responsible for H4K20 mono-methylation (H4K20me1), causes H4K20me1 depletion, accumulation of DNA damage and ATR-dependent cell cycle arrest (Li et al., 2016). H4K16 mono-methylation (H4K16me1) is a recently identified mark involved in 53BP1 recruitment at IR-induced sites of DNA damage (Lu et al., 2019). H4K16me1 is rapidly deposited at damaged sites by the HMT activity of G9a-like protein (GLP) (Figure 1) and this is important for the timely recruitment of 53BP1 to DSBs (Figure 3A). Indeed, unlike H4K16 acetylation, H4K16me1 enhances 53BP1–H4K20me2 interaction at damaged sites. Consistently, GLP knockdown markedly attenuates 53BP1 foci formation after irradiation, leading to impaired NHEJ-mediated repair and decreased cell survival (Lu et al., 2019).

## Lysine Methylation in Histone H2Ax Tail

Recruitment of H2AX and its phosphorylation at serine 139 (γ-H2AX) are crucial regulatory steps in the local activation of DNA-damage repair pathways (Groth et al., 2007; Lukas et al., 2011). Although γ-H2AX deregulation in cancer is well known, the underlying molecular mechanisms and its relationship to other histone modifications are still elusive. It was previously reported that KMT1B (SUV39H2) methylates histone H2AX on lysine 134 (Sone et al., 2014). However, the sequence context of H2AX-K134 does not fit with the specificity of SUV39H2, which generally methylates H3K9. In a subsequent study, *in vitro* methylation reaction assays with SUV39H2 (and its homolog SUV39H1), using H2AX protein and peptides, did not produce any methylation at K134 or any other lysine (Schuhmacher et al., 2016). Nonetheless, these data cannot rule out H2AX methylation by SUV39H2 in cells. In any case, when H2AXK134 is mutated to abolish its methylation, γ-H2AX deposition following DSB-induction is significantly reduced (Sone et al., 2014). The same authors observed lower γ-H2AX levels when DSBs were induced in SUV39H2-deficient cells (Sone et al., 2014). Furthermore, K134-substituted variants of histone H2AX enhanced radio- and chemo-sensitivity of cancer cells (Sone et al., 2014). In conclusion, H2AX methylation plays a role in the regulation of γ-H2AX levels in response to DNA damage.

## Modulation of HMTs and Hdms Expression by IR

**FIGURE 4:**
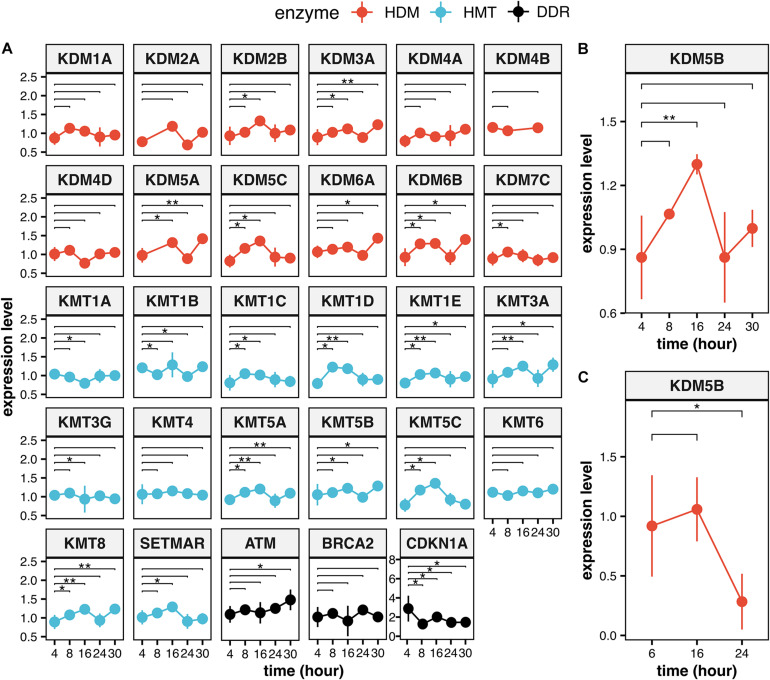
Expression levels of the HMTs and HDMs in response to ionizing radiation. **(A)** Time course of the modulation of all the HMTs and HDMs which are reported on Supplementary Table 1 and of the DDR genes ATM, BRCA2, and CDKN1A after particle irradiation. Data for KDM5B are reported for particle irradiation **(B)** and for X-ray **(C)**. Statistical analysis of experimental data was performed using analysis of variance (ANOVA) followed by multiple comparison Tukey’s test. The data was retrieved from the NCBI Gene Expression Omnibus (GEO) public repository from these datasets: GSE23901, GSE23903, GSE32091, GSE21059, (Ghandhi et al., 2011) GSE23807, GSE30240 (Rashi-Elkeles et al., 2011). All statistical analysis was carried out using native functions of R language version 3.6.3. Expression levels reported are the Log2(IR/control) values. **P < 0.01 and *P < 0.05.

Histone lysine methylation is deeply involved in controlling gene expression in response to environmental stimuli and during cell differentiation (Campos and Reinberg, 2009; Bannister and Kouzarides, 2011; Yun et al., 2011; Venkatesh and Workman, 2015; Cacci et al., 2017). IR is a very powerful inducer of gene expression changes (Amundson et al., 2001; Venkata Narayanan et al., 2017), and some of the induced modulations are maintained in the cell progeny by persistent chromatin modifications, including histone lysine methylation (Ilnytskyy and Kovalchuk, 2011). It is therefore expected that HMTs and HDMs may mediate the response to IR through the transcriptional modulation of DDR related genes impacting on the efficiency of cell response, besides direct chromatin modifications at damaged sites (Penterling et al., 2016). HMTs and HMDs expression levels could be also modulated in response to IR, besides IR-dependent effects on their recruitment to damaged sites. We previously created an interactive database containing data from more than 200 different irradiation conditions (classified by dose and quality of radiation) of different mammalian cell lines and tissues (Chiani et al., 2009). This database (Radiation Genes) can give a useful measure of the IR-dependent transcriptional response of a gene in specific experimental conditions (Fratini et al., 2011; Bufalieri et al., 2012; Licursi et al., 2017). We interrogated the database on the modulation of the HMTs and HDMs involved in DDR, which are reported in Supplementary Table 1. Indeed, we found evidence of IR-dependent upregulation for most of them (15 out of 27) during the first 16 h after particle irradiation (Figure 4A), with 9 genes showing a significant upregulation even after 30 h. The general trend is very similar to that observed for three relevant DDR genes (BRCA2, CDKN1A, and ATM), which we report for comparison. These three genes are among the 20 genes most consistently modulated by IR conditions in the Radiation Genes database (Giusti et al., 2014) and show a significant induction between 4 and 30 h as most of the lysine modification genes. In the framework of this general trend, individual fluctuations can be observed. As an example, KDM5B is generally upregulated 8 h after particle irradiation (0.1 and 0.5 Gy doses), reaches peak expression levels after 16 h and continues to be upregulated at 30 h, albeit at lower levels (Figure 4B). X-ray irradiation does not increase KDM5B levels after 16 h and reduces its levels after 24 h (Figure 4C). X-ray irradiation (2.5 and 5 Gy doses) seems to be generally less effective: the expression changes of genes encoding HMTs and HDMs are lower in intensity in comparison with particle irradiation and tend to disappear at 24 h after irradiation (not shown).

## Open Questions and Perspectives

We learned that the chromatin landscape at damaged genomic sites has a crucial role in determining the choice of the repair pathways and their timing and efficiency. On the one hand, we have an accurate catalog of the histone modifications involved in DDR and of the enzymes performing those modifications. On the other hand, we still have a static view of these modifications. In the case of histone lysine methylation, it is clear that both HTMs and HDMs activities are usually required for an efficient DDR. This means that the signaling pathways impinging on the chromatin at the damaged sites are regulated by a dynamic equilibrium. Analyses of the time course of histone modifications, possibly on cell cycle synchronized cells, are required in order to truly understand these dynamics. More detailed studies are also required to better distinguish among the effects of mono-, di-, and tri-methylation, which often seem to be partially overlapping. Other crucial issues concern the DNA damage specificity of different histone modifications and the crosstalk between them, which we have just started to understand. To address these issues, it is urgent to get a more detailed view of the different chromatin landscapes and histone modifications that are observed in functionally distinct damaged genomic sites. A seminal paper by Clouaire and coworkers presented a comprehensive mapping of histone modifications at DSBs in human cells (Clouaire et al., 2018). This work describes the distribution of several chromatin features at multiple DSBs spread through the genome using ChIP-Seq. The authors could identify NHEJ- and HR-specific chromatin events, which is a first step toward a deep understanding of their functional meaning. This analysis produced some surprising results, such as a reduction of the level of H3K36me2 and H3K79me2 at HR-prone DSBs and an increase of H3K36me3 at the NHEJ-prone DSBs. The authors suggest that these discrepancies with previous observations could arise from the use of different DSB induction methods: DIvA method (AsISI-dependent DSB induction) produces DSBs within or near genes, whereas IR produces DSBs randomly distributed throughout the genome. We now know that DNA lesions in active promoters or coding regions undergo chromatin modifications very different from heterochromatic regions, but high-resolution mapping studies are still limited. We reviewed several studies suggesting that some of the histone modifications observed at damaged sites (i.e., H3K4 demethylation) are required in order to repress local transcription. Recent studies, however, showed that damage-induced long non-coding RNAs (dilncRNAs) synthesized at DSBs by RNA polymerase II are necessary for DDR focus formation, and antisense transcripts against dilncRNAs can inhibit DSB repair (Michelini et al., 2017; Pessina et al., 2019). Thus, conflicts and cross-regulatory events between this damage-induced and highly directional transcription and the potentially pre-existing local transcription could dramatically influence the damage-dependent chromatin changes required in different genomic loci. This remains an entirely open chapter in the study of DDR response. The knowledge about the role of HMTs and HDMs in the response to IR has obvious applications in oncologic radiotherapy. HMTs and HDMs are frequently found to be differentially expressed in human cancer cell lines and their differential expression is often related to increased chemo- and radio-resistance (Bannister and Kouzarides, 2011; Jambhekar et al., 2017; Husmann and Gozani, 2019). Indeed, many studies showed that specific catalytic inhibitors of HMTs or HDMs can lead to radio-sensitization of tumors or cancer cell lines (Gursoy-Yuzugullu et al., 2017; Jambhekar et al., 2017; Bayo et al., 2018; Rath et al., 2018; Cao et al., 2019; Pippa et al., 2019). This field is rapidly expanding in the framework of epigenetic cancer therapy.

## Author Contributions

VL, RN, and EDN conceptualized the review. EDN, GL, VL, and RN contributed to writing the review, constructing the table and figures, and edited the review. VL performed bioinformatic and statistical analysis. VL and RN supervised the work. All authors have read and agreed to the published version of the manuscript.

## Conflict of Interest

The authors declare that the research was conducted in the absence of any commercial or financial relationships that could be construed as a potential conflict of interest.

## Abbreviations

ATM: Ataxia-telangiectasia mutated
DDR: DNA damage response
DIvA: DSB Inducible via AsiSI
DSBs: Double-strand breaks
HDMs: Histone demethylases
HMTs: Histone methyltransferases
HPTMs: Histone post-translational modifications
HR: Homologous recombination
KDMs: Lysine demethylases
KMTs: Lysine methyltransferases
L3MBTL1: Lethal-3-malignant brain tumor-like protein-1
LEDGF: Lens epithelium-derived growth factor
LET: Linear energy transfer
MMS: Methyl methanesulfonate
NHEJ: Non-homologous end joining
SSBs: Single-strand breaks.

## References

[B1] Acs K. Luijsterburg M. S. Ackermann L. Salomons F. A. Hoppe T. Dantuma N. P. (2011). The AAA-ATPase VCP/p97 promotes 53BP1 recruitment by removing L3MBTL1 from DNA double-strand breaks. *Nat. Struct. Mol. Biol.* 18 1345–1350. 10.1038/nsmb.2188 22120668

[B2] Agarwal P. Jackson S. P. (2016). G9a inhibition potentiates the anti-tumour activity of DNA double-strand break inducing agents by impairing DNA repair independent of p53 status. *Cancer Lett.* 380 467–475. 10.1016/j.canlet.2016.07.009 27431310 PMC5011428

[B3] Alagoz M. Katsuki Y. Ogiwara H. Ogi T. Shibata A. Kakarougkas A. (2015). SETDB1, HP1 and SUV39 promote repositioning of 53BP1 to extend resection during homologous recombination in G2 cells. *Nucleic Acids Res.* 43 7931–7944. 10.1093/nar/gkv722 26206670 PMC4652757

[B4] Alonso-de Vega I. Paz-Cabrera M. C. Rother M. B. Wiegant W. W. Checa-Rodríguez C. Hernández-Fernaud J. R. (2020). PHF2 regulates homology-directed DNA repair by controlling the resection of DNA double strand breaks. *Nucleic Acids Res.* 48 4915–4927. 10.1093/nar/gkaa196 32232336 PMC7229830

[B5] Amundson S. A. Bittner M. Meltzer P. Trent J. Fornace A. J. (2001). Induction of gene expression as a monitor of exposure to ionizing radiation. *Radiat. Res.* 156 657–661. 11604088 10.1667/0033-7587(2001)156[0657:iogeaa]2.0.co;2

[B6] Aymard F. Bugler B. Schmidt C. K. Guillou E. Caron P. Briois S. (2014). Transcriptionally active chromatin recruits homologous recombination at DNA double-strand breaks. *Nat. Struct. Mol. Biol.* 21 366–374. 10.1038/nsmb.2796 24658350 PMC4300393

[B7] Ayrapetov M. K. Gursoy-Yuzugullu O. Xu C. Xu Y. Price B. D. (2014). DNA double-strand breaks promote methylation of histone H3 on lysine 9 and transient formation of repressive chromatin. *Proc. Natl. Acad. Sci. U.S.A.* 111 9169–9174. 10.1073/pnas.1403565111 24927542 PMC4078803

[B8] Bannister A. J. Kouzarides T. (2011). Regulation of chromatin by histone modifications. *Cell Res.* 21 381–395. 10.1038/cr.2011.22 21321607 PMC3193420

[B9] Bartek J. Lukas J. (2007). DNA damage checkpoints: from initiation to recovery or adaptation. *Curr. Opin. Cell Biol.* 19 238–245. 10.1016/j.ceb.2007.02.009 17303408

[B10] Bayo J. Tran T. A. Wang L. Peña-Llopis S. Das A. K. Martinez E. D. (2018). Jumonji inhibitors overcome radioresistance in cancer through changes in H3K4 methylation at double-strand breaks. *Cell Rep.* 25 1040–1050e5. 10.1016/j.celrep.2018.09.081 30355483 PMC6245670

[B11] Beucher A. Birraux J. Tchouandong L. Barton O. Shibata A. Conrad S. (2009). ATM and Artemis promote homologous recombination of radiation-induced DNA double-strand breaks in G2. *EMBO J.* 28 3413–3427. 10.1038/emboj.2009.276 19779458 PMC2752027

[B12] Bostelman L. J. Keller A. M. Albrecht A. M. Arat A. Thompson J. S. (2007). Methylation of histone H3 lysine-79 by Dot1p plays multiple roles in the response to UV damage in Saccharomyces cerevisiae. *DNA Repair* 6 383–395. 10.1016/j.dnarep.2006.12.010 17267293

[B13] Boudoures A. L. Pfeil J. J. Steenkiste E. M. Hoffman R. A. Bailey E. A. Wilkes S. E. (2017). A novel histone crosstalk pathway important for regulation of UV-Induced DNA damage repair in saccharomyces cerevisiae. *Genetics* 206 1389–1402. 10.1534/genetics.116.195735 28522541 PMC5500138

[B14] Bromberg K. D. Mitchell T. R. H. Upadhyay A. K. Jakob C. G. Jhala M. A. Comess K. M. (2017). The SUV4-20 inhibitor A-196 verifies a role for epigenetics in genomic integrity. *Nat. Chem. Biol.* 13 317–324. 10.1038/nchembio.2282 28114273

[B15] Bueno M. T. D. Baldascini M. Richard S. Lowndes N. F. (2018). Recruitment of lysine demethylase 2A to DNA double strand breaks and its interaction with 53BP1 ensures genome stability. *Oncotarget* 9 15915–15930. 10.18632/oncotarget.24636 29662616 PMC5882307

[B16] Bufalieri F. Licursi V. D’Antonio M. Castrignanò T. Amendola R. Negri R. (2012). The transcriptional response of mammalian cancer cells to irradiation is dominated by a cell cycle signature which is strongly attenuated in non-cancer cells and tissues. *Int. J. Radiat. Biol.* 88 822–829. 22420862 10.3109/09553002.2012.676230

[B17] Cacci E. Negri R. Biagioni S. Lupo G. (2017). Histone methylation and microRNA-dependent regulation of epigenetic activities in neural progenitor self-renewal and differentiation. *Curr. Top. Med. Chem.* 17 794–807. 10.2174/1568026616666160414124456 27086782

[B18] Campbell S. Ismail I. H. Young L. C. Poirier G. G. Hendzel M. J. (2013). Polycomb repressive complex 2 contributes to DNA double-strand break repair. *Cell Cycle Georget. Tex.* 12 2675–2683. 10.4161/cc.25795 23907130 PMC3865057

[B19] Campos E. I. Reinberg D. (2009). Histones: annotating chromatin. *Annu. Rev. Genet.* 43 559–599. 10.1146/annurev.genet.032608.103928 19886812

[B20] Cao H. Li L. Yang D. Zeng L. Yewei X. Yu B. (2019). Recent progress in histone methyltransferase (G9a) inhibitors as anticancer agents. *Eur. J. Med. Chem.* 179 537–546. 10.1016/j.ejmech.2019.06.072 31276898

[B21] Cao L.-L. Wei F. Du Y. Song B. Wang D. Shen C. (2016). ATM-mediated KDM2A phosphorylation is required for the DNA damage repair. *Oncogene* 35 301–313. 10.1038/onc.2015.81 25823024

[B22] Carvalho S. Vítor A. C. Sridhara S. C. Martins F. B. Raposo A. C. Desterro J. M. P. (2014). SETD2 is required for DNA double-strand break repair and activation of the p53-mediated checkpoint. *eLife* 3:e02482. 10.7554/eLife.02482 24843002 PMC4038841

[B23] Chen R. Zhao W.-Q. Fang C. Yang X. Ji M. (2020). Histone methyltransferase SETD2: a potential tumor suppressor in solid cancers. *J. Cancer* 11 3349–3356. 10.7150/jca.38391 32231741 PMC7097956

[B24] Chen Y. Zhu W.-G. (2016). Biological function and regulation of histone and non-histone lysine methylation in response to DNA damage. *Acta Biochim. Biophys. Sin.* 48 603–616. 10.1093/abbs/gmw050 27217472

[B25] Chernikova S. B. Dorth J. A. Razorenova O. V. Game J. C. Brown J. M. (2010). Deficiency in Bre1 impairs homologous recombination repair and cell cycle checkpoint response to radiation damage in mammalian cells. *Radiat. Res.* 174 558–565. 10.1667/RR2184.1 20738173 PMC2988074

[B26] Chiani F. Iannone C. Negri R. Paoletti D. D’Antonio M. De Meo P. D. (2009). Radiation Genes: a database devoted to microarrays screenings revealing transcriptome alterations induced by ionizing radiation in mammalian cells. *Database* 2009:ba007. 10.1093/database/bap007 20157480 PMC2790304

[B27] Chou D. M. Adamson B. Dephoure N. E. Tan X. Nottke A. C. Hurov K. E. (2010). A chromatin localization screen reveals poly (ADP ribose)-regulated recruitment of the repressive polycomb and NuRD complexes to sites of DNA damage. *Proc. Natl. Acad. Sci. U.S.A.* 107 18475–18480. 10.1073/pnas.1012946107 20937877 PMC2972950

[B28] Clouaire T. Rocher V. Lashgari A. Arnould C. Aguirrebengoa M. Biernacka A. (2018). Comprehensive mapping of histone modifications at DNA double-strand breaks deciphers repair pathway chromatin signatures. *Mol. Cell* 72 250–262e6. 10.1016/j.molcel.2018.08.020 30270107 PMC6202423

[B29] Daugaard M. Baude A. Fugger K. Povlsen L. K. Beck H. Sørensen C. S. (2012). LEDGF (p75) promotes DNA-end resection and homologous recombination. *Nat. Struct. Mol. Biol.* 19 803–810. 10.1038/nsmb.2314 22773103

[B30] Densham R. M. Garvin A. J. Stone H. R. Strachan J. Baldock R. A. Daza-Martin M. (2016). Human BRCA1-BARD1 ubiquitin ligase activity counteracts chromatin barriers to DNA resection. *Nat. Struct. Mol. Biol.* 23 647–655. 10.1038/nsmb 27239795 PMC6522385

[B31] Desouky O. Ding N. Zhou G. (2015). Targeted and non-targeted effects of ionizing radiation. *J. Radiat. Res. Appl. Sci.* 8 247–254. 10.1016/j.jrras.2015.03.003

[B32] D’Oto A. Tian Q.-W. Davidoff A. M. Yang J. (2016). Histone demethylases and their roles in cancer epigenetics. *J. Med. Oncol. Ther.* 1 34–40. 28149961 PMC5279889

[B33] Durante M. (2004). Heavy ion radiobiology for hadrontherapy and space radiation protection. *Radiother. Oncol.* 73 S158–S160. 10.1016/S0167-8140(04)80040-9 15971334

[B34] Fan L. Xu S. Zhang F. Cui X. Fazli L. Gleave M. (2020). Histone demethylase JMJD1A promotes expression of DNA repair factors and radio-resistance of prostate cancer cells. *Cell Death Dis.* 11:214. 10.1038/s41419-020-2405-4 32238799 PMC7113292

[B35] Farooq Z. Banday S. Pandita T. K. Altaf M. (2016). The many faces of histone H3K79 methylation. *Mutat. Res. Rev. Mutat. Res.* 768 46–52. 10.1016/j.mrrev.2016.03.005 27234562 PMC4889126

[B36] Fnu S. Williamson E. A. De Haro L. P. Brenneman M. Wray J. Shaheen M. (2011). Methylation of histone H3 lysine 36 enhances DNA repair by nonhomologous end-joining. *Proc. Natl. Acad. Sci. U.S.A.* 108 540–545. 10.1073/pnas.1013571108 21187428 PMC3021059

[B37] Fradet-Turcotte A. Canny M. D. Escribano-Díaz C. Orthwein A. Leung C. C. Y. Huang H. (2013). 53BP1 is a reader of the DNA-damage-induced H2A Lys 15 ubiquitin mark. *Nature* 499 50–54. 10.1038/nature12318 23760478 PMC3955401

[B38] Fratini E. Licursi V. Artibani M. Kobos K. Colautti P. Negri R. (2011). Dose-dependent onset of regenerative program in neutron irradiated mouse skin. *PLoS One* 6:e19242. 10.1371/journal.pone.0019242 21556364 PMC3083422

[B39] Gao S.-B. Li K.-L. Qiu H. Zhu L.-Y. Pan C.-B. Zhao Y. (2017). Enhancing chemotherapy sensitivity by targeting PcG via the ATM/p53 pathway. *Am. J. Cancer Res.* 7 1874–1883. 28979810 PMC5622222

[B40] Ghandhi S. A. Sinha A. Markatou M. Amundson S. A. (2011). Time-series clustering of gene expression in irradiated and bystander fibroblasts: an application of FBPA clustering. *BMC Genomics* 12:2. 10.1186/1471-2164-12-2 21205307 PMC3022823

[B41] Giusti N. Bufalieri F. Licursi V. Castrignanò T. D’Antonio M. Amendola R. (2014). General features of the transcriptional response of mammalian cells to low- and high-LET irradiation. *Rendiconti Lincei* 25 69–74.

[B42] Gong F. Chiu L.-Y. Cox B. Aymard F. Clouaire T. Leung J. W. (2015). Screen identifies bromodomain protein ZMYND8 in chromatin recognition of transcription-associated DNA damage that promotes homologous recombination. *Genes Dev.* 29 197–211. 10.1101/gad.252189.114 25593309 PMC4298138

[B43] Gong F. Clouaire T. Aguirrebengoa M. Legube G. Miller K. M. (2017). Histone demethylase KDM5A regulates the ZMYND8-NuRD chromatin remodeler to promote DNA repair. *J. Cell Biol.* 216 1959–1974. 10.1083/jcb.201611135 28572115 PMC5496618

[B44] Gong F. Miller K. M. (2019). Histone methylation and the DNA damage response. *Mutat. Res.* 780 37–47. 10.1016/j.mrrev.2017.09.003 31395347 PMC6690396

[B45] Groth A. Rocha W. Verreault A. Almouzni G. (2007). Chromatin challenges during DNA replication and repair. *Cell* 128 721–733. 10.1016/j.cell.2007.01.030 17320509

[B46] Gupta R. Somyajit K. Narita T. Maskey E. Stanlie A. Kremer M. (2018). DNA repair network analysis reveals shieldin as a key regulator of NHEJ and PARP inhibitor sensitivity. *Cell* 173 972–988e23. 10.1016/j.cell.2018.03.050 29656893 PMC8108093

[B47] Gursoy-Yuzugullu O. Carman C. Serafim R. B. Myronakis M. Valente V. Price B. D. (2017). Epigenetic therapy with inhibitors of histone methylation suppresses DNA damage signaling and increases glioma cell radiosensitivity. *Oncotarget* 8 24518–24532. 10.18632/oncotarget.15543 28445939 PMC5421867

[B48] Hausmann M. Wagner E. Lee J.-H. Schrock G. Schaufler W. Krufczik M. (2018). Super-resolution localization microscopy of radiation-induced histone H2AX-phosphorylation in relation to H3K9-trimethylation in HeLa cells. *Nanoscale* 10 4320–4331. 10.1039/c7nr08145f 29443341

[B49] Hendriks I. A. Treffers L. W. Verlaan-de Vries M. Olsen J. V. Vertegaal A. C. O. (2015). SUMO-2 orchestrates chromatin modifiers in response to DNA damage. *Cell Rep.* 10 1778–1791. 10.1016/j.celrep.2015.02.033 25772364 PMC4514456

[B50] Hsiao K.-Y. Mizzen C. A. (2013). Histone H4 deacetylation facilitates 53BP1 DNA damage signaling and double-strand break repair. *J. Mol. Cell Biol.* 5 157–165. 10.1093/jmcb/mjs066 23329852

[B51] Huang J. Dorsey J. Chuikov S. Pérez-Burgos L. Zhang X. Jenuwein T. (2010). G9a and Glp methylate lysine 373 in the tumor suppressor p53. *J. Biol. Chem.* 285 9636–9641. 10.1074/jbc.M109.062588 20118233 PMC2843213

[B52] Huang J. Sengupta R. Espejo A. B. Lee M. G. Dorsey J. A. Richter M. (2007). p53 is regulated by the lysine demethylase LSD1. *Nature* 449 105–108. 10.1038/nature06092 17805299

[B53] Huang Y. Fang J. Bedford M. T. Zhang Y. Xu R.-M. (2006). Recognition of histone H3 lysine-4 methylation by the double tudor domain of JMJD2A. *Science* 312 748–751. 10.1126/science.1125162 16601153

[B54] Husmann D. Gozani O. (2019). Histone lysine methyltransferases in biology and disease. *Nat. Struct. Mol. Biol.* 26 880–889. 10.1038/s41594-019-0298-7 31582846 PMC6951022

[B55] Huyen Y. Zgheib O. Ditullio R. A. Gorgoulis V. G. Zacharatos P. Petty T. J. (2004). Methylated lysine 79 of histone H3 targets 53BP1 to DNA double-strand breaks. *Nature* 432 406–411. 10.1038/nature03114 15525939

[B56] Ilnytskyy Y. Kovalchuk O. (2011). Non-targeted radiation effects-an epigenetic connection. *Mutat. Res.* 714 113–125. 10.1016/j.mrfmmm.2011.06.014 21784089

[B57] Jacquet K. Fradet-Turcotte A. Avvakumov N. Lambert J.-P. Roques C. Pandita R. K. (2016). The TIP60 complex regulates bivalent chromatin recognition by 53BP1 through Direct H4K20me binding and H2AK15 acetylation. *Mol. Cell* 62 409–421. 10.1016/j.molcel.2016.03.031 27153538 PMC4887106

[B58] Jambhekar A. Anastas J. N. Shi Y. (2017). Histone lysine demethylase inhibitors. *Cold Spring Harb. Perspect. Med.* 7:a026484. 10.1101/cshperspect.a026484 28049654 PMC5204329

[B59] Kang J.-Y. Kim J.-Y. Kim K.-B. Park J. W. Cho H. Hahm J. Y. (2018). KDM2B is a histone H3K79 demethylase and induces transcriptional repression via sirtuin-1-mediated chromatin silencing. *FASEB J.* 32 5737–5750. 10.1096/fj.201800242R 29763382

[B60] Kang J.-Y. Park J. W. Hahm J. Y. Jung H. Seo S.-B. (2020). Histone H3K79 demethylation by KDM2B facilitates proper DNA replication through PCNA dissociation from chromatin. *Cell Prolif.* 53:e12920. 10.1111/cpr.12920 33029857 PMC7653264

[B61] Kari V. Raul S. K. Henck J. M. Kitz J. Kramer F. Kosinsky R. L. (2019). The histone methyltransferase DOT1L is required for proper DNA damage response, DNA repair, and modulates chemotherapy responsiveness. *Clin. Epigenetics* 11:4. 10.1186/s13148-018-0601-1 30616689 PMC6323691

[B62] Katagi H. Louis N. Unruh D. Sasaki T. He X. Zhang A. (2019). Radiosensitization by histone H3 demethylase inhibition in diffuse intrinsic pontine glioma. *Clin. Cancer Res.* 25 5572–5583. 10.1158/1078-0432.CCR-18-3890 31227500 PMC6744979

[B63] Khoury-Haddad H. Guttmann-Raviv N. Ipenberg I. Huggins D. Jeyasekharan A. D. Ayoub N. (2014). PARP1-dependent recruitment of KDM4D histone demethylase to DNA damage sites promotes double-strand break repair. *Proc. Natl. Acad. Sci. U.S.A.* 111 E728–E737. 10.1073/pnas.1317585111 24550317 PMC3932863

[B64] Khurana S. Kruhlak M. J. Kim J. Tran A. D. Liu J. Nyswaner K. (2014). A macrohistone variant links dynamic chromatin compaction to BRCA1-dependent genome maintenance. *Cell Rep.* 8 1049–1062. 10.1016/j.celrep.2014.07.024 25131201 PMC4154351

[B65] Lazzaro F. Sapountzi V. Granata M. Pellicioli A. Vaze M. Haber J. E. (2008). Histone methyltransferase Dot1 and Rad9 inhibit single-stranded DNA accumulation at DSBs and uncapped telomeres. *EMBO J.* 27 1502–1512. 10.1038/emboj.2008.81 18418382 PMC2328446

[B66] Lee J. Thompson J. R. Botuyan M. V. Mer G. (2008). Distinct binding modes specify the recognition of methylated histones H3K4 and H4K20 by JMJD2A-tudor. *Nat. Struct. Mol. Biol.* 15 109–111. 10.1038/nsmb1326 18084306 PMC2211384

[B67] Li Q. Shi L. Gui B. Yu W. Wang J. Zhang D. (2011). Binding of the JmjC demethylase JARID1B to LSD1/NuRD suppresses angiogenesis and metastasis in breast cancer cells by repressing chemokine CCL14. *Cancer Res.* 71 6899–6908. 10.1158/0008-5472.CAN-11-1523 21937684

[B68] Li X. Liu L. Yang S. Song N. Zhou X. Gao J. (2014). Histone demethylase KDM5B is a key regulator of genome stability. *Proc. Natl. Acad. Sci. U.S.A.* 111 7096–7101. 10.1073/pnas.1324036111 24778210 PMC4024858

[B69] Li Y. Armstrong R. L. Duronio R. J. MacAlpine D. M. (2016). Methylation of histone H4 lysine 20 by PR-Set7 ensures the integrity of late replicating sequence domains in Drosophila. *Nucleic Acids Res.* 44 7204–7218. 10.1093/nar/gkw333 27131378 PMC5009726

[B70] Licursi V. Cestelli Guidi M. Del Vecchio G. Mannironi C. Presutti C. Amendola R. (2017). Leptin induction following irradiation is a conserved feature in mammalian epithelial cells and tissues. *Int. J. Radiat. Biol.* 93 947–957. 28593811 10.1080/09553002.2017.1339918

[B71] Lu X. Tang M. Zhu Q. Yang Q. Li Z. Bao Y. (2019). GLP-catalyzed H4K16me1 promotes 53BP1 recruitment to permit DNA damage repair and cell survival. *Nucleic Acids Res.* 47 10977–10993. 10.1093/nar/gkz897 31612207 PMC6868394

[B72] Luger K. Dechassa M. L. Tremethick D. J. (2012). New insights into nucleosome and chromatin structure: an ordered state or a disordered affair? *Nat. Rev. Mol. Cell Biol.* 13 436–447. 10.1038/nrm3382 22722606 PMC3408961

[B73] Lukas J. Lukas C. Bartek J. (2011). More than just a focus: the chromatin response to DNA damage and its role in genome integrity maintenance. *Nat. Cell Biol.* 13 1161–1169. 10.1038/ncb2344 21968989

[B74] Mallette F. A. Mattiroli F. Cui G. Young L. C. Hendzel M. J. Mer G. (2012). RNF8- and RNF168-dependent degradation of KDM4A/JMJD2A triggers 53BP1 recruitment to DNA damage sites. *EMBO J.* 31 1865–1878. 10.1038/emboj.2012.47 22373579 PMC3343333

[B75] Mavragani I. V. Nikitaki Z. Kalospyros S. A. Georgakilas A. G. (2019). Ionizing radiation and complex DNA damage: from prediction to detection challenges and biological significance. *Cancers* 11:1789. 10.3390/cancers11111789 31739493 PMC6895987

[B76] McGinty R. K. Tan S. (2015). Nucleosome structure and function. *Chem. Rev.* 115 2255–2273. 10.1021/cr500373h 25495456 PMC4378457

[B77] Michelini F. Pitchiaya S. Vitelli V. Sharma S. Gioia U. Pessina F. (2017). Damage-induced lncRNAs control the DNA damage response through interaction with DDRNAs at individual double-strand breaks. *Nat. Cell Biol.* 19 1400–1411. 10.1038/ncb3643 29180822 PMC5714282

[B78] Mirza-Aghazadeh-Attari M. Mohammadzadeh A. Yousefi B. Mihanfar A. Karimian A. Majidinia M. (2019). 53BP1: a key player of DNA damage response with critical functions in cancer. *DNA Repair* 73 110–119. 10.1016/j.dnarep.2018.11.008 30497961

[B79] Misteli T. Soutoglou E. (2009). The emerging role of nuclear architecture in DNA repair and genome maintenance. *Nat. Rev. Mol. Cell Biol.* 10 243–254. 10.1038/nrm2651 19277046 PMC3478884

[B80] Mocavini I. Pippa S. Licursi V. Paci P. Trisciuoglio D. Mannironi C. (2019). JARID1B expression and its function in DNA damage repair are tightly regulated by miRNAs in breast cancer. *Cancer Sci.* 110 1232–1243. 10.1111/cas.13925 30588710 PMC6447846

[B81] Mosammaparast N. Kim H. Laurent B. Zhao Y. Lim H. J. Majid M. C. (2013). The histone demethylase LSD1/KDM1A promotes the DNA damage response. *J. Cell Biol.* 203 457–470. 10.1083/jcb.201302092 24217620 PMC3824007

[B82] O’Hagan H. M. Mohammad H. P. Baylin S. B. (2008). Double strand breaks can initiate gene silencing and SIRT1-dependent onset of DNA methylation in an exogenous promoter CpG island. *PLoS Genet.* 4:e1000155. 10.1371/journal.pgen.1000155 18704159 PMC2491723

[B83] O’Hagan H. M. Wang W. Sen S. Destefano Shields C. Lee S. S. Zhang Y. W. (2011). Oxidative damage targets complexes containing DNA methyltransferases, SIRT1, and polycomb members to promoter CpG Islands. *Cancer Cell* 20 606–619. 10.1016/j.ccr.2011.09.012 22094255 PMC3220885

[B84] Panier S. Boulton S. J. (2014). Double-strand break repair: 53BP1 comes into focus. *Nat. Rev. Mol. Cell Biol.* 15 7–18. 10.1038/nrm3719 24326623

[B85] Pedersen M. T. Helin K. (2010). Histone demethylases in development and disease. *Trends Cell Biol.* 20 662–671. 10.1016/j.tcb.2010.08.011 20863703

[B86] Pei H. Zhang L. Luo K. Qin Y. Chesi M. Fei F. (2011). MMSET regulates histone H4K20 methylation and 53BP1 accumulation at DNA damage sites. *Nature* 470 124–128. 10.1038/nature09658 21293379 PMC3064261

[B87] Peng B. Wang J. Hu Y. Zhao H. Hou W. Zhao H. (2015). Modulation of LSD1 phosphorylation by CK2/WIP1 regulates RNF168-dependent 53BP1 recruitment in response to DNA damage. *Nucleic Acids Res.* 43 5936–5947. 10.1093/nar/gkv528 25999347 PMC4499147

[B88] Peng J. C. Karpen G. H. (2009). Heterochromatic genome stability requires regulators of histone H3 K9 methylation. *PLoS Genet.* 5:e1000435. 10.1371/journal.pgen.1000435 19325889 PMC2654965

[B89] Penterling C. Drexler G. A. Böhland C. Stamp R. Wilke C. Braselmann H. (2016). Depletion of histone demethylase Jarid1A resulting in histone hyperacetylation and radiation sensitivity does not affect DNA double-strand break repair. *PLoS One* 11:e0156599. 10.1371/journal.pone.0156599 27253695 PMC4890786

[B90] Pessina F. Giavazzi F. Yin Y. Gioia U. Vitelli V. Galbiati A. (2019). Functional transcription promoters at DNA double-strand breaks mediate RNA-driven phase separation of damage-response factors. *Nat. Cell Biol.* 21 1286–1299. 10.1038/s41556-019-0392-4 31570834 PMC6859070

[B91] Peters A. H. O’Carroll D. Scherthan H. Mechtler K. Sauer S. Schöfer C. (2001). Loss of the Suv39h histone methyltransferases impairs mammalian heterochromatin and genome stability. *Cell* 107 323–337. 10.1016/s0092-8674(01)00542-6 11701123

[B92] Pfister S. X. Ahrabi S. Zalmas L.-P. Sarkar S. Aymard F. Bachrati C. Z. (2014). SETD2-dependent histone H3K36 trimethylation is required for homologous recombination repair and genome stability. *Cell Rep.* 7 2006–2018. 10.1016/j.celrep.2014.05.026 24931610 PMC4074340

[B93] Pippa S. Mannironi C. Licursi V. Bombardi L. Colotti G. Cundari E. (2019). Small molecule inhibitors of KDM5 histone demethylases increase the radiosensitivity of breast cancer cells overexpressing JARID1B. *Mol. Basel Switz.* 24:1739. 10.3390/molecules24091739 31060229 PMC6540222

[B94] Polo S. E. Jackson S. P. (2011). Dynamics of DNA damage response proteins at DNA breaks: a focus on protein modifications. *Genes Dev.* 25 409–433. 10.1101/gad.2021311 21363960 PMC3049283

[B95] Price B. D. (2017). KDM5A demethylase: erasing histone modifications to promote repair of DNA breaks. *J. Cell Biol.* 216 1871–1873. 10.1083/jcb.201705005 28572116 PMC5496629

[B96] Rashi-Elkeles S. Elkon R. Shavit S. Lerenthal Y. Linhart C. Kupershtein A. (2011). Transcriptional modulation induced by ionizing radiation: p53 remains a central player. *Mol. Oncol.* 5 336–348. 10.1016/j.molonc.2011.06.004 21795128 PMC5528315

[B97] Rath B. H. Waung I. Camphausen K. Tofilon P. J. (2018). Inhibition of the histone H3K27 demethylase UTX enhances tumor cell radiosensitivity. *Mol. Cancer Ther.* 17 1070–1078. 10.1158/1535-7163.MCT-17-1053 29483212 PMC5932086

[B98] Rona G. Roberti D. Yin Y. Pagan J. K. Homer H. Sassani E. (2018). PARP1-dependent recruitment of the FBXL10-RNF68-RNF2 ubiquitin ligase to sites of DNA damage controls H2A.Z loading. *eLife* 7:e38771. 10.7554/eLife.38771 29985131 PMC6037479

[B99] Rondinelli B. Gogola E. Yücel H. Duarte A. A. van de Ven M. van der Sluijs R. (2017). EZH2 promotes degradation of stalled replication forks by recruiting MUS81 through histone H3 trimethylation. *Nat. Cell Biol.* 19 1371–1378. 10.1038/ncb3626 29035360

[B100] Schuhmacher M. K. Kudithipudi S. Jeltsch A. (2016). Investigation of H2AX methylation by the SUV39H2 protein lysine methyltransferase. *FEBS Lett.* 590 1713–1719. 10.1002/1873-3468.12216 27177470

[B101] Shanbhag N. M. Rafalska-Metcalf I. U. Balane-Bolivar C. Janicki S. M. Greenberg R. A. (2010). ATM-dependent chromatin changes silence transcription in cis to DNA double-strand breaks. *Cell* 141 970–981. 10.1016/j.cell.2010.04.038 20550933 PMC2920610

[B102] Shi Y. Lan F. Matson C. Mulligan P. Whetstine J. R. Cole P. A. (2004). Histone demethylation mediated by the nuclear amine oxidase homolog LSD1. *Cell* 119 941–953. 10.1016/j.cell.2004.12.012 15620353

[B103] Simon J. A. Kingston R. E. (2009). Mechanisms of polycomb gene silencing: knowns and unknowns. *Nat. Rev. Mol. Cell Biol.* 10 697–708. 10.1038/nrm2763 19738629

[B104] Sone K. Piao L. Nakakido M. Ueda K. Jenuwein T. Nakamura Y. (2014). Critical role of lysine 134 methylation on histone H2AX for γ-H2AX production and DNA repair. *Nat. Commun.* 5:5691. 10.1038/ncomms6691 25487737 PMC4268694

[B105] Sun Y. Jiang X. Xu Y. Ayrapetov M. K. Moreau L. A. Whetstine J. R. (2009). Histone H3 methylation links DNA damage detection to activation of the tumour suppressor Tip60. *Nat. Cell Biol.* 11 1376–1382. 10.1038/ncb1982 19783983 PMC2783526

[B106] Takahashi A. Imai Y. Yamakoshi K. Kuninaka S. Ohtani N. Yoshimoto S. (2012). DNA damage signaling triggers degradation of histone methyltransferases through APC/C(Cdh1) in senescent cells. *Mol. Cell* 45 123–131. 10.1016/j.molcel.2011.10.018 22178396

[B107] Tang J. Cho N. W. Cui G. Manion E. M. Shanbhag N. M. Botuyan M. V. (2013). Acetylation limits 53BP1 association with damaged chromatin to promote homologous recombination. *Nat. Struct. Mol. Biol.* 20 317–325. 10.1038/nsmb.2499 23377543 PMC3594358

[B108] Tellier M. Chalmers R. (2019). Human SETMAR is a DNA sequence-specific histone-methylase with a broad effect on the transcriptome. *DNA Repair* 80 26–35. 10.1016/j.dnarep.2019.06.006 30329085 PMC6326780

[B109] Tsukada Y. Fang J. Erdjument-Bromage H. Warren M. E. Borchers C. H. Tempst P. (2006). Histone demethylation by a family of JmjC domain-containing proteins. *Nature* 439 811–816. 10.1038/nature04433 16362057

[B110] Vacík T. Laðinoviæ D. Raška I. (2018). KDM2A/B lysine demethylases and their alternative isoforms in development and disease. *Nucl. Austin Tex.* 9 431–441. 10.1080/19491034.2018.1498707 30059280 PMC7000146

[B111] Vakoc C. R. Sachdeva M. M. Wang H. Blobel G. A. (2006). Profile of histone lysine methylation across transcribed mammalian chromatin. *Mol. Cell. Biol.* 26 9185–9195. 10.1128/MCB.01529-06 17030614 PMC1698537

[B112] Venkata Narayanan I. Paulsen M. T. Bedi K. Berg N. Ljungman E. A. Francia S. (2017). Transcriptional and post-transcriptional regulation of the ionizing radiation response by ATM and p53. *Sci. Rep.* 7:43598. 10.1038/srep43598 28256581 PMC5335570

[B113] Venkatesh S. Workman J. L. (2015). Histone exchange, chromatin structure and the regulation of transcription. *Nat. Rev. Mol. Cell Biol.* 16 178–189. 10.1038/nrm3941 25650798

[B114] Vidanes G. M. Bonilla C. Y. Toczyski D. P. (2005). Complicated tails: histone modifications and the DNA damage response. *Cell* 121 973–976. 10.1016/j.cell.2005.06.013 15989948

[B115] Wardman P. (2009). The importance of radiation chemistry to radiation and free radical biology (The 2008 Silvanus Thompson Memorial Lecture). *Br. J. Radiol.* 82 89–104. 10.1259/bjr/60186130 19168690

[B116] Wei S. Li C. Yin Z. Wen J. Meng H. Xue L. (2018). Histone methylation in DNA repair and clinical practice: new findings during the past 5-years. *J. Cancer* 9 2072–2081. 10.7150/jca.23427 29937925 PMC6010677

[B117] Williams K. Christensen J. Rappsilber J. Nielsen A. L. Johansen J. V. Helin K. (2014). The histone lysine demethylase JMJD3/KDM6B is recruited to p53 bound promoters and enhancer elements in a p53 dependent manner. *PLoS One* 9:e96545. 10.1371/journal.pone.0096545 24797517 PMC4010471

[B118] Wojtala M. Dąbek A. Rybaczek D. Śliwińska A. Świderska E. Słapek K. (2019). Silencing lysine-specific histone demethylase 1 (LSD1) causes increased HP1-positive chromatin, stimulation of DNA repair processes, and dysregulation of proliferation by Chk1 phosphorylation in human endothelial cells. *Cells* 8:1212. 10.3390/cells8101212 31591366 PMC6829479

[B119] Wu Z. Lee S. T. Qiao Y. Li Z. Lee P. L. Lee Y. J. (2011). Polycomb protein EZH2 regulates cancer cell fate decision in response to DNA damage. *Cell Death Differ.* 18 1771–1779. 10.1038/cdd.2011.48 21546904 PMC3190113

[B120] Xu W. Zhou B. Zhao X. Zhu L. Xu J. Jiang Z. (2018). KDM5B demethylates H3K4 to recruit XRCC1 and promote chemoresistance. *Int. J. Biol. Sci.* 14 1122–1132. 10.7150/ijbs.25881 29989047 PMC6036731

[B121] Young L. C. McDonald D. W. Hendzel M. J. (2013). Kdm4b histone demethylase is a DNA damage response protein and confers a survival advantage following γ-irradiation. *J. Biol. Chem.* 288 21376–21388. 10.1074/jbc.M113.491514 23744078 PMC3774405

[B122] Yun M. Wu J. Workman J. L. Li B. (2011). Readers of histone modifications. *Cell Res.* 21 564–578. 10.1038/cr.2011.42 21423274 PMC3131977

[B123] Zhang C. Hong Z. Ma W. Ma D. Qian Y. Xie W. (2013). *Drosophila* UTX coordinates with p53 to regulate ku80 expression in response to DNA damage. *PLoS One* 8:e78652. 10.1371/journal.pone.0078652 24265704 PMC3827076

[B124] Zhang J. He P. Xi Y. Geng M. Chen Y. Ding J. (2015). Down-regulation of G9a triggers DNA damage response and inhibits colorectal cancer cells proliferation. *Oncotarget* 6 2917–2927. 10.18632/oncotarget.2784 25595900 PMC4413627

[B125] Zhu B. Chen S. Wang H. Yin C. Han C. Peng C. (2018). The protective role of DOT1L in UV-induced melanomagenesis. *Nat. Commun.* 9:259. 10.1038/s41467-017-02687-7 29343685 PMC5772495

