# Corrigendum: The Role of Histone Lysine Methylation in the Response of Mammalian Cells to Ionizing Radiation

**DOI:** 10.3389/fgene.2022.896771

**Published:** 2022-04-13

**Authors:** Elena Di Nisio, Giuseppe Lupo, Valerio Licursi, Rodolfo Negri

**Affiliations:** ^1^ Department of Biology and Biotechnology Charles Darwin, Sapienza University of Rome, Rome, Italy; ^2^ Institute of Molecular Biology and Pathology, National Research Counsil (IBPM-CNR), Rome, Italy

**Keywords:** DNA damage, ionizing radiation, DNA repair, HPTMs, histone methylation

In the original article, there was a mistake in “Figure 3” as published. There was an error in a representation of the histone tail of H3 in the lysine 4 residue in Figures 3A,B: we added K4 label on the H4 tail instead of H3 one. The corrected “Figure 3” appears below.

**FIGURE 3:**
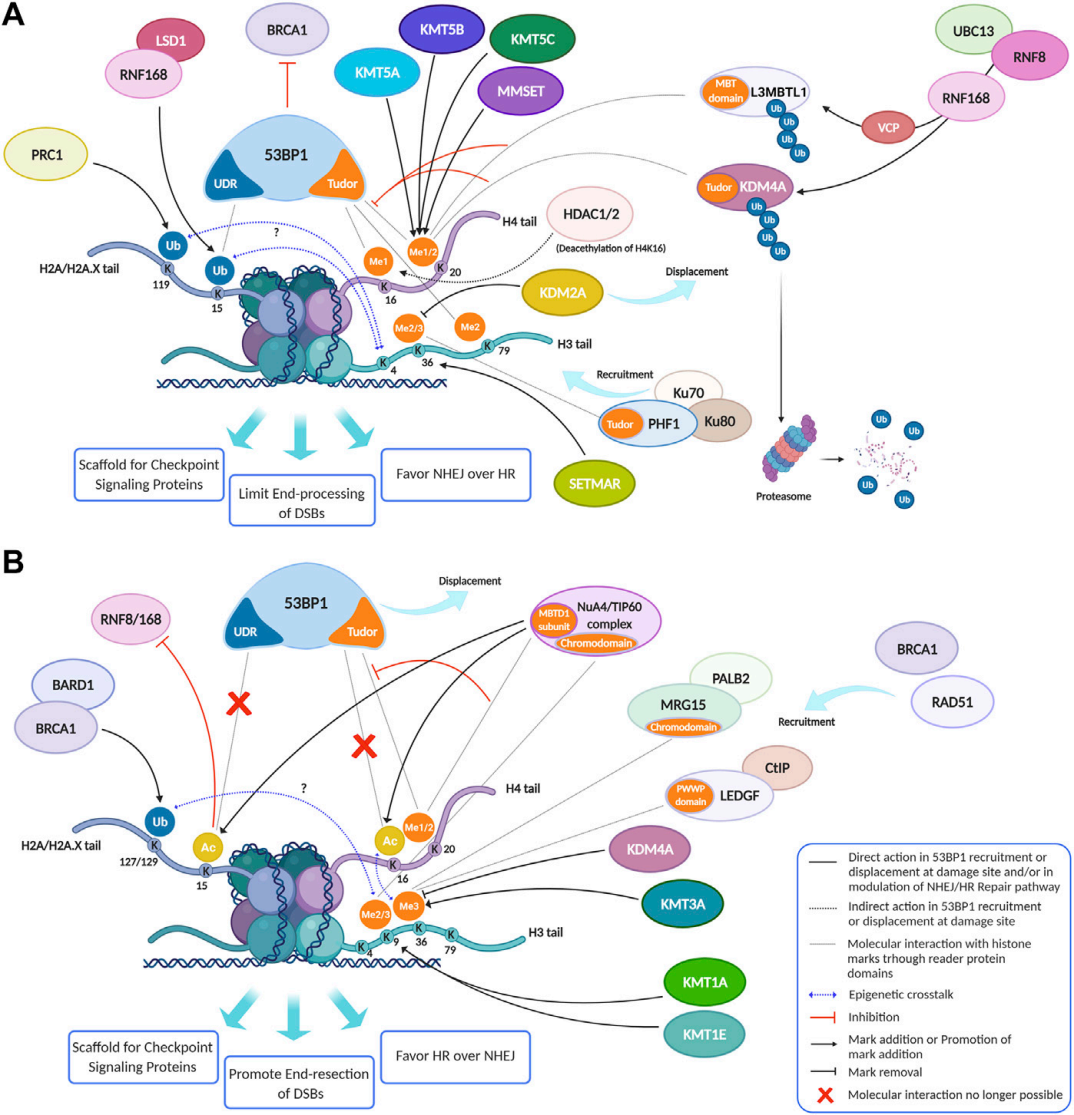
Histone modifications, dynamically added or removed around the DNA damage site, constitute an integrated signal for the DSBs repair pathway choice. **(A)** 53BP1 is a master regulator of DNA double-strand break repair pathway choice toward NHEJ and its binding to chromatin is mainly regulated by specific histone modifications, comprising H2A ubiquitylation and H3 and H4 methylation. Indeed, specific HPTMs can generate a docking site for 53BP1, which binds the ubiquitinated H2A/H2A.X K13/15/119 through its UDR motif and H4K20me2, H4K16me1, and H3K79me2 through its tandem Tudor domain. Therefore, despite H4K20me2 being the main histone mark involved in 53BP1 recruitment, other marks may contribute to it. For example, the deacetylation of H4K16 by HDAC1/2 indirectly promotes the 53BP1 recruitment and the NHEJ over HR, because it makes possible the methylation of this lysine residue. Under normal conditions, the H4K20me2 mark is masked by various interacting proteins, including L3MBTL1 and KDM4A/JMJD2A. Upon DNA DSBs induction, the ATM phosphorylation cascade promotes the recruitment of the E3 ubiquitin ligase RNF8 to the DNA damage site, which, together with the E2 ubiquitin ligase UBC13, promotes the relocation of the E3 ubiquitin ligase RNF168 to the DNA break sites. LSD1 may favor the 53BP1 recruitment interacting with RNF168 and/or thanks to a possible epigenetic cross-talk between H3K4 demethylation and H2A ubiquitylation. RNF168 catalyzes in turn the H2A K13/15 mono-ubiquitination and the poly-ubiquitination of L3MBTL1 and KDM4A, leading to RNF8-dependent degradation of KDM4A and to VCP-mediated and ubiquitin-dependent removal of L3MBTL1 from chromatin, favoring the unmasking of H4K20me2 and the loading of 53BP1 around the damaged site. LSD1 can also disadvantage 53BP1 recruitment, negatively regulating the interaction between p53 and 53BP1 (not shown). After DSB induction by IR, the H3K36me2 markedly increases, improving the association of early NHEJ factors such as Ku70/Ku80 and NBS1 at the damage site. Once activated, ATM (not shown) may negatively regulate KDM2A chromatin-binding capacity, favoring its displacement, which favors an increase of H3K36 di-methylation mark near the DNA damage sites by SETMAR. H3K36me3 can favor the retention of the NHEJ-associated factor PHF1, which is recruited in a Ku70/Ku80 dependent way and which supports an open chromatin for the efficient DNA repair. In contrast to H3K36me2, H3K36me3 regulates both NHEJ and HR, probably depending on the relative abundance of its reader factors and/or by the chromatin context. In this regard, pre-existing transcription-associated and cell cycle-regulated histone modifications together with those damage-induced regulate the DNA repair pathways. **(B)** The pre-existing H3K36me3 mark at actively transcribed gene bodies favors DNA repair by HR pathway choice: in human cells, both LEDGF (Lens epithelium-derived growth factor), *via* its PWWP reader domain, and MRG15, *via* its chromodomain, can bind H3K36me3, promoting the recruitment of CtIP and PALB2, two crucial factors for the DNA end-resection and strand invasion, respectively. Moreover, mutually exclusive histone modifications can contribute to tip the balance between HR and NHEJ in DSB repair pathway choice: a switch between ubiquitylation and acetylation on H2AK15 and H4K16 can favor HR over NHEJ. The human NuA4/TIP60 acetyltransferase complex is able to inhibit the 53BP1 recruitment both through this mechanism and by targeting the same H4K20me2 mark, by binding H4K20me with the MBT (malignant brain tumor) domain of MBTD1, its stable subunit. Moreover, the human NuA4/TIP60 acetyltransferase complex promotes the repositioning of 53BP1 through other mechanisms, such as the acetylation of H2A/H2AX K13/15 that blocks the recruitment of RNF8/168 and through the acetylation of H4K16 that impedes the methylation of this lysin residue, limiting the recruitment of 53BP1. KDM4A, besides masking H3K20me2 and limiting the 53BP1 recruitment thus inhibiting the NHEJ pathway, negatively regulates the DDR also removing H3K36me3 mark thus inhibiting the HR pathway. Conversely, KMT3A favors the HR through the methylation of H3K36. KMT1E favors the HR though the methylation of H3K9. Indeed, the H3K9me2 mark is involved in the recruitment of NuA4/TIP60 acetyltransferase complex, and H3K9me3 is involved in the recruitment of BARD1/BRCA1 Ub ligase activity on H2AK127 favoring the DNA end-resection, the repositioning of 53BP1 and thus the HR. Created using BioRender.

In the original article, there was a mistake in the legend of “Figure 3A” as published. In the row number 4 of the legend there is a typo: H3K79me3 instead of H3K79me2. The correct legend appears below.

The authors apologize for this error and state that this does not change the scientific conclusions of the article in any way. The original article has been updated.

